# Supplementary material for: Exploring Consumer Behavior and Preferences in Welfare-Friendly Pork Breeding: A Multivariate Analysis
Source: Foods. 2023 Aug 10;12(16):3014. doi: 10.3390/foods12163014 (PMC10453549; doi:10.3390/foods12163014)
Supplement: Supplementary file 1 [file foods-12-03014-s001.zip › foods-2496209-supplementary.pdf]

Questo questionario è oggetto di una ricerca scientifica condotta dal Dipartimento di Scienze Veterinarie in sinergia con il Dipartimento di Economia dell'Università degli Studi di Messina al fine di valutare la sensibilità dell'opinione pubblica verso il benessere animale, con particolare attenzione alle pratiche mutilanti condotte nelle aziende suinicole. La sua partecipazione è totalmente anonima e volontaria

1. Sesso
2. Età
3. Qual è il suo titolo di studio?
4. Nazionalità?
5. Comune e Provincia di residenza?
6. Stato civile?
7. Attuale condizione lavorativa?
8. A quale fascia di reddito (al netto delle tasse) appartiene la sua famiglia?<br>(La risposta è facoltativa tuttavia le ricordo che il questionario è anonimo)
9. Quante persone compongono il suo nucleo familiare (lei compreso)?
10. È credente?
11. Di quale delle seguenti categorie fa parte?
12. Quanto è importante per lei il consumo di carne suina?
13. Quante volte alla settimana consuma carne suina (carne fresca)?
14. Quante volte alla settimana consuma salumi di carne suina?
15. Indichi di seguito le ragioni per le quali preferisce carne suina rispetto ad altre tipologie di carne:
16. Quali di questi elementi considera più importanti nell'acquisto della carne suina: attribuire un punteggio da 0 a 10 (0 minima importanza, 10 massima importanza):
17. Si reputa sensibile al benessere degli animali da reddito?
18. Ha avuto delle esperienze lavorative nel campo dell'allevamento del bestiame?
19. Quali sono le sue fonti di informazione relative agli allevamenti dei suini?
20. Prima di oggi conosceva il problema dell'odore di verro o boar taint?
21. Conosce quali sono le mutilazioni consentite dalla normativa vigente?
22. In considerazione delle informazioni da noi fornite reputa giusta la castrazione dei suinetti?
23. In considerazione delle informazioni da noi fornite reputa giusta l'applicazione delle pratiche mutilanti?
24. In considerazione dalle informazioni da noi fornite a quale pratica mutilante si reputa favorevole? [il mozzamento della coda]
25. In considerazione delle informazioni da noi fornite a quale metodo di castrazione si reputa favorevole? [Immunocastrazione]
26. Stante che le pratiche mutilanti (il mozzamento della coda, la troncatura degli incisivi e l'applicazione dell'anello al naso) possono essere evitate se i suini
27. Se NO per quale motivo? (il questionario riprende alla domanda n. 31)
28. Quanto pagherebbe di più la carne di suini allevati con metodi alternativi alla castrazione chirurgica senza anestesia?
29. Quanto pagherebbe di più la carne di suini allevati con metodiche aziendali che non ricorrono alle mutilazioni per implementare il benessere animale?
30. Per quale motivo sarebbe disposto a pagare di più per l'acquisto di carne di suini castrati con metodi alternativi alla castrazione chirurgica senza anestesia?
31. Per quale motivo sarebbe disposto a pagare di più per l'acquisto di carne di suini allevati con metodiche aziendali che non ricorrono alle mutilazioni

32. Come giudica un allevamento di suini che rispetta il benessere dell'animale ad elevati standard rispetto ad un allevamento che pratica le mutilazioni
33. Ritiene che gli allevamenti di suini che rispettano il benessere dell'animale siano adeguatamente pubblicizzati e sostenuti?
